# The influence of mid-latitude storm tracks on hot, cold, dry and wet extremes

**DOI:** 10.1038/srep17491

**Published:** 2015-12-11

**Authors:** Jascha Lehmann, Dim Coumou

**Affiliations:** 1 Potsdam Institute for Climate Impact Research, 14412 Potsdam, Germany; 2 University of Potsdam, Am Neuen Palais 10, 14469 Potsdam, Germany.

## Abstract

Changes in mid-latitude circulation can strongly affect the number and intensity of extreme weather events. In particular, high-amplitude quasi-stationary planetary waves have been linked to prolonged weather extremes at the surface. In contrast, analyses of fast-traveling synoptic-scale waves and their direct influence on heat and cold extremes are scarce though changes in such waves have been detected and are projected for the 21st century. Here we apply regression analyses of synoptic activity with surface temperature and precipitation in monthly gridded observational data. We show that over large parts of mid-latitude continental regions, summer heat extremes are associated with low storm track activity. In winter, the occurrence of cold spells is related to low storm track activity over parts of eastern North America, Europe, and central- to eastern Asia. Storm tracks thus have a moderating effect on continental temperatures. Pronounced storm track activity favors monthly rainfall extremes throughout the year, whereas dry spells are associated with a lack thereof. Trend analyses reveal significant regional changes in recent decades favoring the occurrence of cold spells in the eastern US, droughts in California and heat extremes over Eurasia.

In 2014, California suffered a severe drought^1^ whereas the northeastern US experienced record-breaking Arctic cold in winter^2^. In the same year, droughts in the northern province of Liaoning – China’s northern bread basket – threatened crop yields while the UK was affected by severe floodings^3^. This seeming accumulation of weather extremes demonstrates the importance of understanding the physical mechanisms driving climate variability in the Northern Hemisphere mid-latitudes at interannual to multi-decadal time scales^4^,^5^. Anomalous large-scale atmospheric circulations are often directly linked to the occurrence of regional temperature and precipitation extremes^6^,^7^,^8^,^9^,^10^,^11^. It is thus critically important to understand how different types of waves in the mid-latitudes influence surface weather and extremes^2^,^8^,^12^,^13^,^14^.

There is substantial evidence that amplified quasi-stationary planetary waves favor particular regional weather extremes^14^,^15^ including hot, cold, dry and wet extremes. Such waves are associated with high-pressure blocking systems which cause persistent and therefore often extreme weather like for example during the Russian heat wave in summer 2010^16^,^17^,^18^. Storm track variability also has a direct influence on regional weather conditions, and their strength and position might change under future climate change^19^,^20^,^21^. Recent studies have shown that the Californian drought was linked to a lack of storm activity^1^ whereas the UK flooding was due to an unusual clustering and persistence of storms^3^. Studies on synoptic-scale wave activity and its association with blocking have mainly focused on wintertime circulation and the influence of shifting storm tracks on blocking frequencies^22^,^23^. Notably, Dong *et al.*^24^ report less atmospheric blocking (defined by a blocking index) in the UK and northwestern Europe when more summer storms travel across these regions. High- and low frequency waves are linked, with large-scale quasi-stationary waves influencing the location and activity of storm tracks which in turn help to maintain the large-scale flow via eddy transports^22^,^25^,^26^. Thus, findings derived from storm tracks will also relate to the larger scale flow including quasi-stationary waves.

In the mid-latitudes, synoptic storms bring moist air from the ocean to the land and in regions like Europe and North America they account for over 70% of total precipitation^27^. Extreme rainfall is thus often associated with strong storm activity^28^,^29^. Storm activity is also likely to affect temperature extremes^30^. In summer – when near-surface temperatures are lower over oceans than over land – storms transport cool air from the oceans to continental regions. A decrease in summer storm activity could thus lead to the build-up of hot and dry conditions over the continents^31^. During winter this effect might reverse as sea surface temperatures are higher than land temperatures. Storm activity is thus likely to have a moderating effect on continental temperatures and hence changes in storm tracks could affect not only precipitation and wind extremes but also heat waves and cold spells.

The position and strength of storm track activity is strongly coupled to large-scale teleconnection patterns like the North Atlantic Oscillation (NAO)^9^,^10^,^32^. The NAO index is often defined as the mean winter (December to March) standardized air pressure difference between the Azores High and the Icelandic Low with positive index values indicating that air pressure is anomalously high over the Azores and anomalously low over Iceland and vice versa for negative index values. During positive NAO phases the North Atlantic storm track shifts northwards associated with mild and wet conditions over northern Europe and colder and drier conditions over southern Europe and the Mediterranean^7^,^8^,^33^. During negative NAO phases, the westerly winds weaken associated with more frequent blocking conditions over Greenland^32^,^34^. In these situations cold air from the Arctic can be dragged to northern Europe leading to anomalously cold winter temperatures over the UK and Scandinavia^35^. There exists a counterpart for the NAO in summer called the summer North Atlantic Oscillation (SNAO)^24^,^33^,^36^. In its positive phase, this pattern is characterized by low air pressures over Greenland and high pressures over northern Europe^33^. The SNAO strongly influences summertime variability of temperature, precipitation and cloudiness over Europe and parts of North America and the Sahel zone through changes in the position of the North Atlantic storm track^36^. During a negative phase of SNAO (with relatively high pressure conditions over Greenland and low pressure anomalies over the British Isles and Scandinavia) the North Atlantic storm track shifts southwards and extends downstream into central Europe associated with more storms and thus more precipitation over the UK and northwest Europe and less rain over southern Europe^24^. In contrast, high-index SNAO summers are related to warm, dry and cloud-free conditions over northwest Europe and much of southern Scandinavia and anomalously cool, wet and cloudier conditions over southern Europe and the Mediterranean^36^,^33^.

The (S)NAO index is usually determined through first empirical orthogonal function analysis of sea level pressure in the North Atlantic sector and thus represents a single number that captures the dominant variability pattern on a near-hemispheric scale. Composite analysis of positive and negative (S)NAO phases thus illustrates the regional changes associated with the dominant variability pattern only and might thus provide little information on local extremes. Most studies on NAO simulations in climate change scenarios focus on winter only^9^. Based on these studies, no clear statements about NAO changes under CO_2_ doubling can be made since some models project an upward trend and others a downward trend. In contrast, robust changes in summer storm track activity have been observed^31^ and are projected for the 21st century under continued global warming^21^,^31^. In particular, climate models project a substantial weakening of summer storm track activity over essentially all of the mid-latitudes^21^ and a poleward shift and downstream extension into Europe during winter^20^,^21^,^37^,^38^ which will likely alter the intensity and frequency of surface weather extremes in these regions.

Here we take an alternative approach and study the link between storm track activity and surface weather extremes directly. We analyze extremes in land surface temperature and precipitation using monthly ECWMF ERA-Interim data^39^. We thus consider extremes associated with prolonged heat waves, cold spells and wet or dry periods. To analyze the effect of storm track activity on temperature extremes over continents, we separate between summer (May-September) and winter (November-February) season since their effect is expected to be opposite (see Methods). Storms are associated with moist air independent of season and hence the analysis of precipitation extremes is applied to the full year. Storm track activity is represented by monthly eddy kinetic energy (EKE), which is computed by bandpass filtering daily wind field data thereby extracting wind speed variability on 2.5–6 days associated with synoptic-scale (eddy) activity^21^,^40^. Since quasi-stationary waves have much lower frequencies they are excluded from the computation of EKE. Likewise, short-lived thunderstorms which typically form in summer have much higher wave frequencies and are thus not considered in this study. We apply quantile regressions^41^ between EKE and temperature or precipitation at each individual grid point. This method allows us to analyze how the tails of the EKE distribution (10^th^ and 90^th^-percentile) as well as the median (50^th^-percentile) are linked to regional surface weather extremes, rather than just estimating the effect of changes in the mean. Linear regression analyses are applied between EKE and geopotential height anomaly fields at individual grid points to assess how synoptic eddy activity is related to blocking anticyclonic flow. All analyses presented in the main manuscript are shown for the 850 mb pressure level. Sensitivity analyses performed at the 500 mb pressure level lead to similar results (see Methods).

## Results

### Quantile regression analysis between EKE and temperature and precipitation

**Figure 1:**
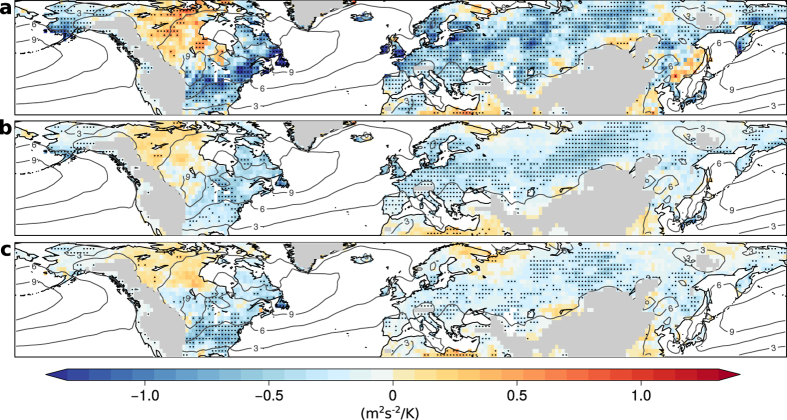
Slope of quantile regressions between anomalies of EKE and temperature in summer. Regression slopes are shown for the 90^th^-percentile (**a**), 50^th^-percentile (**b**), and 10^th^-percentile (**c**) with the middle panel being similar to Fig. 4 from Coumou *et al.*^31^. Stippling indicates significance at the 5%-level and grey contour lines denote EKE climatology in summer. Land regions higher than 1 km have been masked. All maps are created using the open source software R.

Significant negative correlations between anomalies in EKE and land surface temperature in summer can be found over storm track relevant land regions, i.e. North America and Eurasia (Fig. 1a, for EKE climatology see contours in Fig. 1a and Figs S6 and S7 in Supplementary Information (SI)). In these regions, EKE in the 10% hottest summer months is reduced by 20–40% compared to summer climatology (Fig. S8a). Most regression slopes are significant and steepest for the 90^th^-percentile and smaller in magnitude for the 50^th^ and 10^th^-percentile, respectively (Fig. 1, S10a). These different magnitudes imply that the hottest summer months are always associated with low EKE, whereas anomalously cool summer months are associated with larger EKE and a broader range in possible EKE values. Significant regression slopes are evident in all three quantile regressions emphasizing the strong inverse link between EKE and temperatures in summer.

**Figure 2:**
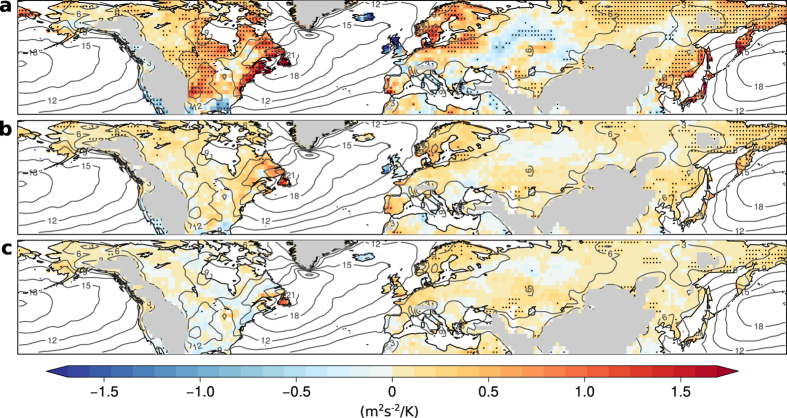
Slope of quantile regressions between anomalies of EKE and temperature in winter. Regression slopes are shown for the 90^th^-percentile (**a**), 50^th^-percentile (**b**), and 10^th^-percentile (**c**). Stippling indicates significance at the 5%-level and grey contour lines denote EKE climatology in winter. Land regions higher than 1 km have been masked. All maps are created using the open source software R.

In winter, significant positive regression slopes in the 90^th^-percentile are found over large parts of North America and Eurasia where mean EKE in the 10% coldest months decreased by 20–40% compared to local winter climatology (Fig. 2a, Fig. S8b). Over North America, we see a dipole pattern with significant negative correlations west of the Rocky Mountains and significant positive correlations to the east. In Scandinavia and most regions in central and east Asia low EKE during winter is significantly correlated with low temperatures. Locally, this relationship is opposite over the UK and parts of western Russia. Regression slopes are again largest for the high quantiles, whereas slopes for the 10^th^-percentile become almost flat (Fig. 2c, Fig. S10b). This implies that in regions with positive correlations the coldest winter months are always associated with low EKE but warm winter months can be associated with a broad range of EKE values.

**Figure 3:**
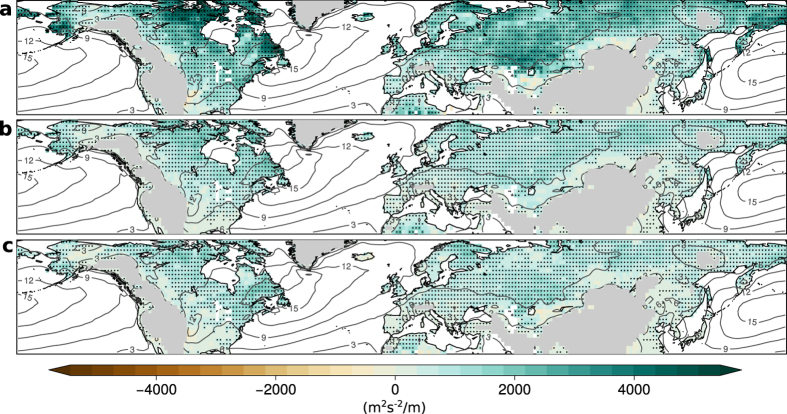
Slope of quantile regressions between anomalies of EKE and precipitation in all calendar months. Regression slopes are shown for the 90^th^-percentile (**a**), 50^th^-percentile (**b**), and 10^th^-percentile (**c**). Stippling indicates significance at the 5%-level and grey contour lines denote annual EKE climatology. Land regions higher than 1 km have been masked. All maps are created using the open source software R.

Monthly precipitation extremes are related to significantly higher EKE in essentially all mid-latitude continental regions (Fig. 3). In particular, we find significant positive correlations between EKE and precipitation with mean EKE significantly increasing by 20–50% in the 10% wettest months compared to annual climatology (Fig. S8c). Consistently, the 10% driest months were associated with an approximately 20–30% drop in EKE (Fig. S8d). Regression slopes are generally larger for the upper quantiles (Fig. S10c) but with significant correlations in all quantiles. We repeated the analysis with precipitation records derived from rain gauge and satellite data taken from the Global Precipitation Climatology Project (GPCP)^42^. We find quantitatively similar regression patterns which confirm the robust link between EKE and precipitation (Figs S14 and S15).

### Linear regression analysis between EKE and geopotential height anomaly fields

**Figure 4:**
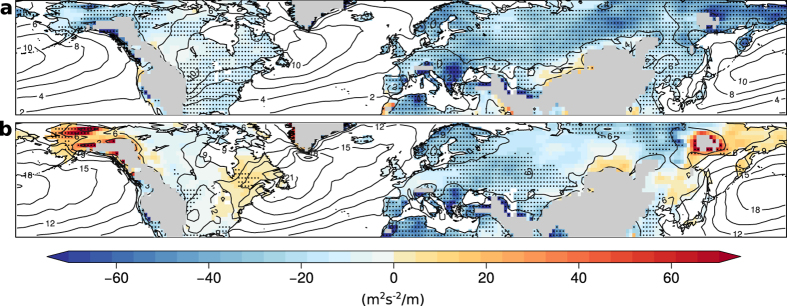
Slope of linear regression between anomalies of EKE and GPH. Regression slopes are shown for summer (**a**) and winter (**b**). Stippling indicates significance at the 5%-level and grey contour lines denote EKE climatology in the corresponding season. Land regions higher than 1 km have been masked. All maps are created using the open source software R.

EKE is significantly anti-correlated with monthly geopotential height (GPH) over essentially all storm track relevant land regions in both summer and winter season (Fig. 4). This implies that low EKE is associated with positive geopotential height anomalies and hence high pressure systems. In winter, an inverse link with low EKE being associated with negative GPH anomalies (i.e. low pressure systems) is shown over Alaska, eastern North America and the coastal regions of eastern Asia (Fig. 4b). However, also in winter, significant negative correlations between EKE and GPH are dominant over most continental land regions.

### Discussion

Our findings show that monthly rainfall extremes are associated with strong storm track activity and dry extremes with a lack thereof. Storms bring moderate weather conditions to the continents and therefore also modulate temperature extremes. This implies that in summer wet spells are associated with cool months whereas dry periods come along with warm months. In winter, we find an inverse link with higher storm track activity being associated with warmer and wetter conditions and less storm track activity being associated with colder and drier months. In agreement with our results, Trenberth and Shea^30^ show that summers over the continents in both hemispheres tend to be either cold and wet or hot and dry, but not any other combination. In winter they find a positive link between temperature and precipitation at high latitudes, implying that here warm winters are generally wet, whereas cold winters are rather dry. In the Northern Hemisphere, these high latitude regions largely overlap with storm track affected regions in agreement with our findings.

The (S)NAO largely influences the position of storm tracks over the North Atlantic affecting weather conditions particularly over Europe and the Mediterranean^9^,^10^,^32^. In winter, a positive NAO results in a poleward shift of the North Atlantic storm track associated with anomalously warm and wet conditions over northern Europe and colder and drier conditions over southern Europe and the Mediterranean^7^,^8^,^33^ consistent with negative regression slopes between temperature and EKE and positive regression slopes between precipitation and EKE seen over these regions. Composite plots of negative and positive SNAO phases reveal that warm and dry conditions in summer are located over regions characterized by blocking anticyclonic flow^24^,^36^,^33^ and thus low storm track activity. This is also seen in our regression maps which indicate that low summertime EKE is significantly linked to positive geopotential height anomalies and thus warm summer temperatures and low precipitation over Europe. The intensified precipitation over the Mediterranean region in positive SNAO phases cannot be explained by SNAO itself as its characteristic pressure anomalies are too far north to directly influence the inflow of maritime air into southern Europe^33^. Over this region, we find significant correlations between EKE and both temperature and precipitation which explain the observed precipitation pattern by changes in storm track activity.

The moderating affect of storm track activity on near-surface temperature is more pronounced in summer than in winter. This could be related to soil moisture-temperature feedbacks which are important in summer^43^. Low summer EKE implies low rainfall and high temperatures which both have a drying effect on the soil. Once the soil has dried out, no more heat can be converted into latent heat by evaporation and temperatures can spike^44^,^45^. There is thus a direct and indirect way how EKE can influence summer temperatures. Moreover, we have not addressed the effect of wind direction on temperature anomalies which is likely more important in winter^46^ and thus could be a reason for weaker correlations found between EKE and winter temperatures. Further, we show that low EKE is significantly correlated with anomalously large geopotential heights over storm track relevant land regions. We thus argue that low EKE creates favorable conditions for atmospheric blocking and hence persistent weather over continental land at least in summer. In agreement, Dong *et al.*^24^ report an increased storm activity over northern Europe associated with less blocking in this region.

**Figure 5:**
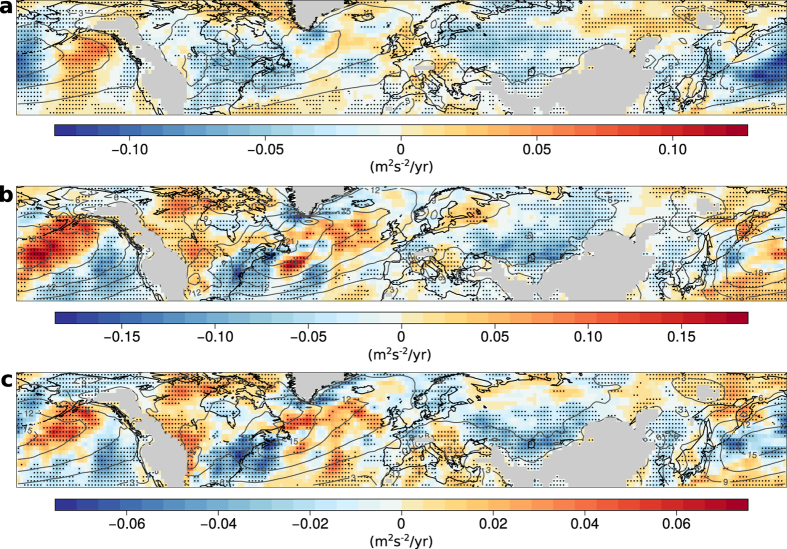
Trends in EKE. Trends are calculated for the time period 1979–2014 in summer (**a**), winter (**b**), and annually (**c**). Grey contour lines denote EKE climatology in the corresponding season and land regions higher than 1 km have been masked. All maps are created using the open source software R.

The presented correlations between storm track activity and surface weather conditions in terms of temperature and precipitation do not allow for direct conclusions about causality. However, extratropical storms mostly form and develop over the oceans (with exception of the US Rockies)^47^ and therefore it is reasonable to assume that over land storm track activity is influencing surface conditions and not the other way around^7^,^32^. Nevertheless, causal interpretations for the cyclogenesis region to the lee of the Rockies have to be treated with caution. Regionally, significant trends in EKE are detected (Fig. 5) which thus might have contributed to observed weather extremes. Notably ~78% of the mid-latitude continental land regions (35° N–65° N) have experienced downward trends in summertime EKE (Fig. 5a). Our results suggest that weakening of summertime EKE over Eurasia and eastern North America created favorable conditions for observed heat extremes like the European heat wave in 2003 or 2010 in Russia and likewise the high temperature anomalies over central to eastern North America in 2012 setting new all-time record high temperatures in multiple states along the US east coast^48^.

These trend analyses are in agreement with Horton *et al.*^12^ who report upward trends in the frequency and persistence of summertime anticyclonic circulation patterns since 1979 over eastern US, Europe and western Asia. They show that the observed trends in circulation contributed significantly to high temperature extremes in these regions. Consistently, these regions also show pronounced downward trends in summertime EKE (Fig. 5a or Fig. S16 for better comparison). Coumou *et al.*^31^ proposed a physical mechanism to explain the observed weakening in summer circulation over recent decades. The observed hemispheric-mean reduction in summer EKE is associated with a decline in the zonal flow, in a similar way as projected by CMIP5 climate models. They argue that the downward trend has likely been influenced by the reduction in the equator-to-pole temperature gradient in response to Arctic amplification. Under a high emission scenario, CMIP5 models project further weakening in the summertime EKE which can be explained by a decrease in the vertical wind shear^21^. Here we report that EKE mostly has a moderating effect on surface weather conditions, notably in summer. Regional weakening of storm activity in recent years thus likely favored the occurrence of summer heat extremes over storm track relevant land regions.

In winter, our results suggest that regional downward trends in EKE likely favored the occurrence of observed cold extremes. Eastern North America has been affected by severe cold spells in recent years^2^,^49^,^50^ which is in agreement with a pronounced reduction in storm track activity found for this region. California also experienced a significant reduction in EKE in winter which likely contributed to the observed periods of extreme droughts. Note, that in California the rainy season falls into the months between October and April whereas the summer months are generally dry. In contrast, central North America has seen an increase in wintertime EKE. Upward trends in EKE are also found over the western North Pacific and the North Atlantic with pronounced regions of negative trends in between. These wave-like patterns over the North American sector, which are also seen in the mid-troposphere (Fig. S18), might reflect changes in the position of the jet. Wang *et al.*^1^ showed that in winter large-scale wave energy in the western North Pacific intensified the high-pressure system offshore California which lead to extreme dry conditions over California in 2013/14. Consistently, we find that downward trends in wintertime EKE in this region are associated with an intensification of high-pressure systems and hence persistent anticyclonic flow (Figs 4b and 5b). Cyclones traveling eastwards across the North Pacific would be deflected and curl around this high-pressure system consistent with the pronounced increase in EKE observed to the northwest and northeast of the ridge.

Another explanation for the observed correlation between synoptic-scale activity and surface weather extremes could be a common (third) driver which influences both transient waves and surface conditions. Large-scale quasi stationary waves might act as such a common driver. They are closely connected to fast-moving transients^22^,^25^,^26^ and amplification of these waves have been shown to favor heat waves in western North America and central Asia, cold spells in eastern North America, wet spells in western Asia and droughts in central North America, Europe, and central Asia^14^. Consistently, we find strong correlations between EKE and temperature or rainfall extremes over similar regions. Especially, we see a similar sensitivity of eastern North America to cold spells and a general link between higher than normal summer temperatures and low EKE.

Synoptic eddies bring weather variability on 2–6 day timescales. We find that changes in the synoptic eddy activity have a moderating affect on near-surface temperatures and are positively correlated with rainfall over mid-latitude continental regions. Low EKE is associated with anticyclonic flow and persistent weather conditions which can lead to extremes on monthly timescales. This is shown for cold spells but is especially pronounced for summertime heat extremes and droughts. We argue that substantial trends in EKE over recent years created favorable conditions for the occurrence of observed extreme weather events in the US and Eurasia and that projected robust changes in EKE under future global warming^20^,^21^,^37^,^38^ will alter the occurrence of both temperature and rainfall extremes over storm track relevant continental regions.

## Method

### Data

Monthly-mean near-surface temperature (2 m above surface), precipitation and geopotential height for the time period 1979–2014 were taken from the ECMWF ERA-Interim data sets^39^. In addition, monthly-mean precipitation derived from a combination of rain gauges and satellites were taken from the GPCP v2.2 data set^42^. Monthly-mean EKE was computed from bandpass filtered^21^,^40^,^51^ daily zonal (u’) and meridional wind speeds (v’) at 850 mb with EKE = 0.5 × (u’^2^ + v’^2^). The v’ component is very well linked to storm tracks and the u’ component less so. However, applying the analysis to v’^2^ instead of EKE leads to similar results (Figs S3 to S5). The analysis was also repeated with EKE computed at mid-troposphere (500 mb) which resulted in similar observed correlations with temperature but weaker correlations with precipitation (SI text S2), indicating that precipitation is sensitive to surface-near circulation changes. Similarly, correlations between EKE and geopotential height anomaly fields are stronger at the lower troposphere than at 500 mb (Fig. S17). The daily wind field data was taken from the ECMWF ERA-Interim data set, for the same time period and with the same grid resolution of 1.5° × 1.5°. For each calendar month and grid point the local climatological mean was subtracted from the grid-box value to compute time series of anomalies for EKE, temperature, precipitation, and geopotential height. Subsequently, all time series were linearly detrended over the full time period, except for the precipitation data set because of its non-Gaussian distribution. However, analysis of linearly detrended precipitation time series gave essentially the same results.

### Seasonality

Mid-latitude storms bring maritime air from the ocean to continental regions. Depending on the season this air can have a cooling (in summer) or warming (in winter) effect. For the analysis of temperature extremes we thus define two seasons; a summer season (May-June-July-August-September) – where ocean surface temperatures are lower than land surface temperatures – and a winter season (November-December-January-February) – where ocean surface temperatures are higher than on land. For the analysis of precipitation extremes we tested and showed that the seasonal cycle has no significant effect (SI text S1). Hence, in the paper results are shown for the full year.

### Quantile regression

Quantile regressions were applied between detrended EKE anomalies and detrended temperature anomalies or precipitation anomalies at each individual grid point. We computed regression slopes for the 10^th^, 50^th^, and 90^th^-percentiles in order to analyze how changes in the tails of the EKE distribution are related to changes in temperature or precipitation. The applied method is described in detail in Koenker and d’Orey^41^. Significant regression slopes were defined at the 5%-level based on confidence intervals computed from a rank test^52^.

### Linear regression

Linear regression analyses were applied at each grid point between detrended EKE anomalies and detrended anomalies of geopotential height with both variables taken at 850 mb. Significant regression slopes were defined at the 5%-level.

### Trend analysis

Linear trends were computed for seasonal-mean EKE at each grid point with significant trends defined at the 5%-level.

### EKE change in hot, cold, dry and wet months

For each grid point and month, the 10% most positive (or most negative) detrended temperature anomalies or precipitation anomalies were chosen as a representation of hot, cold, dry and wet months, respectively. This corresponds to approximately 14–43 data points depending on the number of months in the given season. Hence, 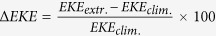 is the change in percent between the mean EKE in these “extreme” months (*EKE*_*extr.*_) and the climatology of EKE (*EKE*_*clim.*_) in a particular month at a certain grid point. To test whether *EKE*_*extr.*_ is significantly different from *EKE*_*clim.*_ we applied a two-sample t-test as well as a Kolmogorow-Smirnow test. We defined significance at the 5%-level with both methods giving similar results (Figs S8 and S9). Note, that we focus on meteorological extremes, i.e. largest deviations from climatology. This definition of extremes could in principle be different to specifying extremes based on absolute values. However, since we analyze heat extremes in summer and cold extremes in winter we expect this difference to be minor.

## Additional Information

**How to cite this article**: Lehmann, J. and Coumou, D. The influence of mid-latitude storm tracks on hot, cold, dry and wet extremes. *Sci. Rep.* **5**, 17491; doi: 10.1038/srep17491 (2015).

## Supplementary Material

Supplementary Information
