# Supplementary material for: The influence of mid-latitude storm tracks on hot, cold, dry and wet extremes
Source: Sci Rep. 2015 Dec 11;5:17491. doi: 10.1038/srep17491 (PMC4675986; doi:10.1038/srep17491)
Supplement: Supplementary Information [file srep17491-s1.pdf]

Supplementary Materials for:

The influence of mid-latitude storm track activity on hot, cold, dry and wet extremes

Jascha Lehmann* and Dim Coumou
*Corresponding author. E-mail: jlehmann@pik-potsdam.de

S1: Seasonal analysis of extremes

The analysis of temperature extremes requires a seasonal approach, since the effect of storms on land surface temperatures will be different between summer and winter season. This is demonstrated in Fig. S1a,b which show the seasonal cycles of mid-latitude mean land surface and sea surface temperature. In summer, the oceans warm slower than the continents, but they also keep the heat longer than land when it gets colder in winter. Consequently, the seasonal cycle is less pronounced over the oceans than over land with sea surface temperature lagging behind land surface temperature by about one month. Two distinct seasons can be derived from this mechanism: the summer season (May-September) where land surface temperature is above sea surface temperature and the winter season (November-February) with the opposite behavior. More precisely, mid-latitude storms bring maritime conditions from the Atlantic to Europe and from the Pacific to North America. Seasonal surface temperatures over the Atlantic and Europe (Fig. S1c) and over the Pacific and North America (Fig. S1d) reveal the same delay between sea and land surface temperatures as observed for the mid-latitudes in general.

The influence of mid-latitude storms on precipitation does not show a significant dependence on the season. Consistently, regression patterns between EKE and precipitation anomalies look essentially identical between summer and winter season (Fig. S2). Only the magnitude of the regression slopes differs as precipitation is generally stronger in winter. In our study we thus use all calendar months for the analysis of precipitation extremes.

S2: Sensitivity analysis of altitude

To test the sensitivity of our regression analysis to EKE at different pressure levels, we repeated the regression of temperature and precipitation anomalies with EKE at 500 mb. Results show that in summer EKE at 500 mb is represented by a much more zonal flow compared to EKE at 850 mb (see Fig. S6-S7). This is due to less orographic friction at higher altitudes. However,

resulting slopes of quantile regression analyses reveal similar patterns at both pressure levels, i.e. negative correlations over essentially all mid-latitude continental regions with most slopes being statistically significant at the 5%-level (Fig. S11). Similarly, we find consistent regression patterns between both pressure levels in winter (Fig. S12).

Correlation analysis between precipitation and EKE seems to be more sensitive to the pressure level at which we compute EKE. Whereas the correlation between precipitation and EKE is generally positive and statistically significant at the lower troposphere, regression slopes are very small in magnitude and mostly non-significant at mid-troposphere (Fig. S13).

S3: Additional figures

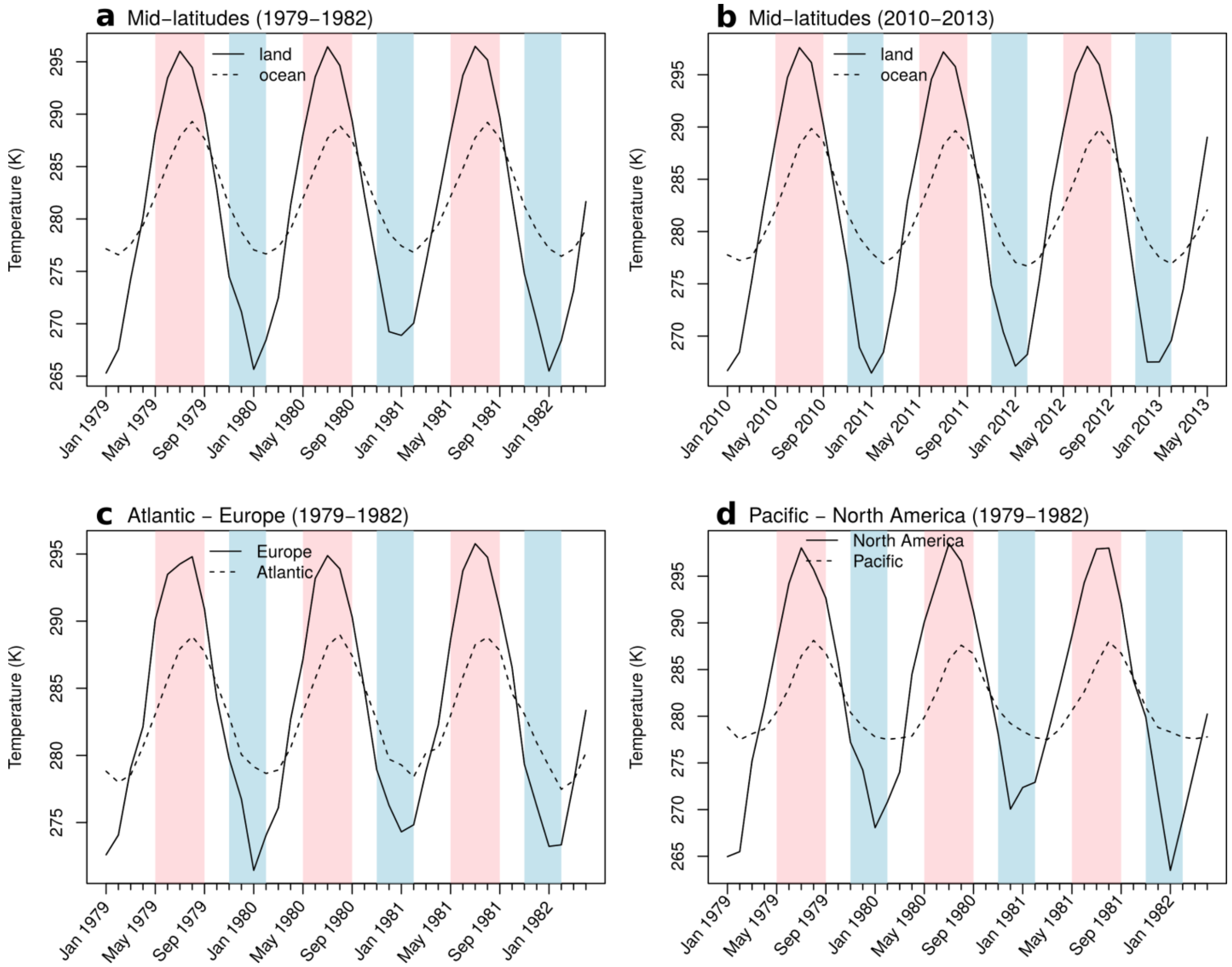

Fig. S1. Seasonal cycle of surface temperatures taken from the ERA-Interim data set at 1000 mb. Temperature curves are shown for land (solid line) and ocean (dashed line) averages in different regions: mid-latitudes in 1979-1982 (**a**), mid-latitudes in 2010-2013 (**b**), Atlantic and Europe (**c**), and Pacific and North America (**d**) in 1979-1982. We define a summer season (May-

September, pink bars) where land surface temperatures are above sea surface temperatures and vice versa for the winter season (November-February, blue bars).

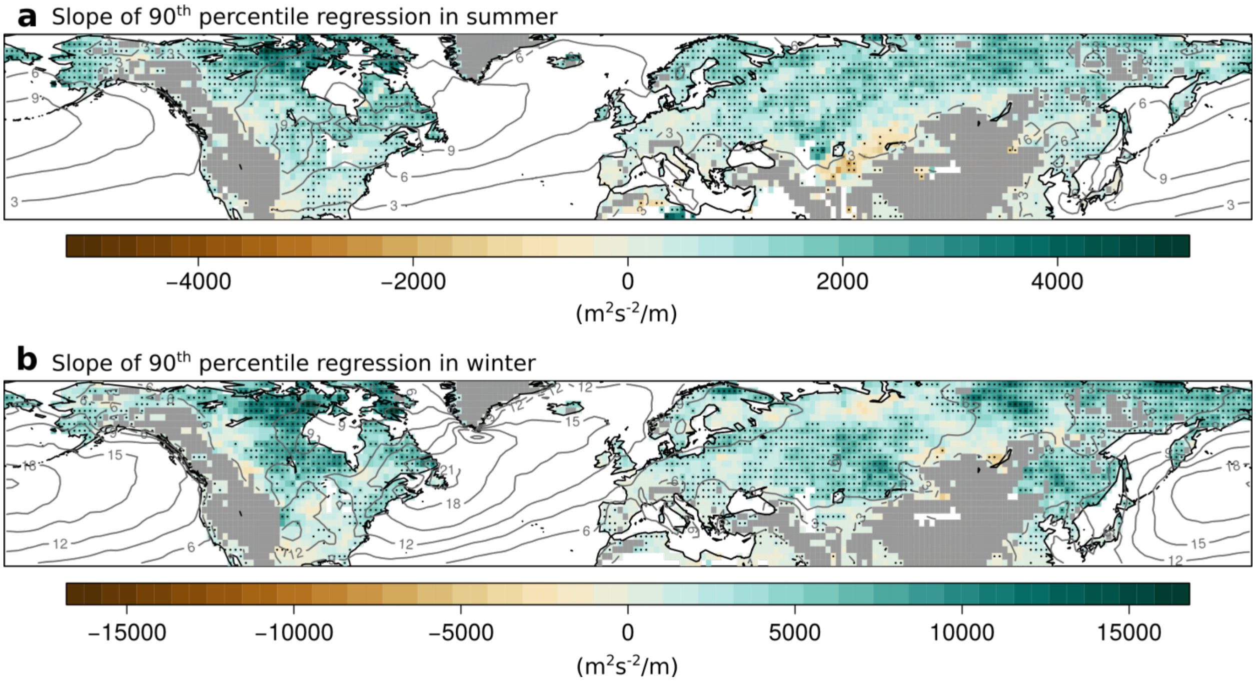

Fig. S2. Slope of 90th-percentile regressions between anomalies in EKE and precipitation in different seasons. Results are similar between summer (**a**) and winter season (**b**). Stippling indicates significance at the 5%-level. Grey contour lines indicate EKE climatology of the given season. Land regions higher than 1 km have been masked. All maps are created using the open source software R.

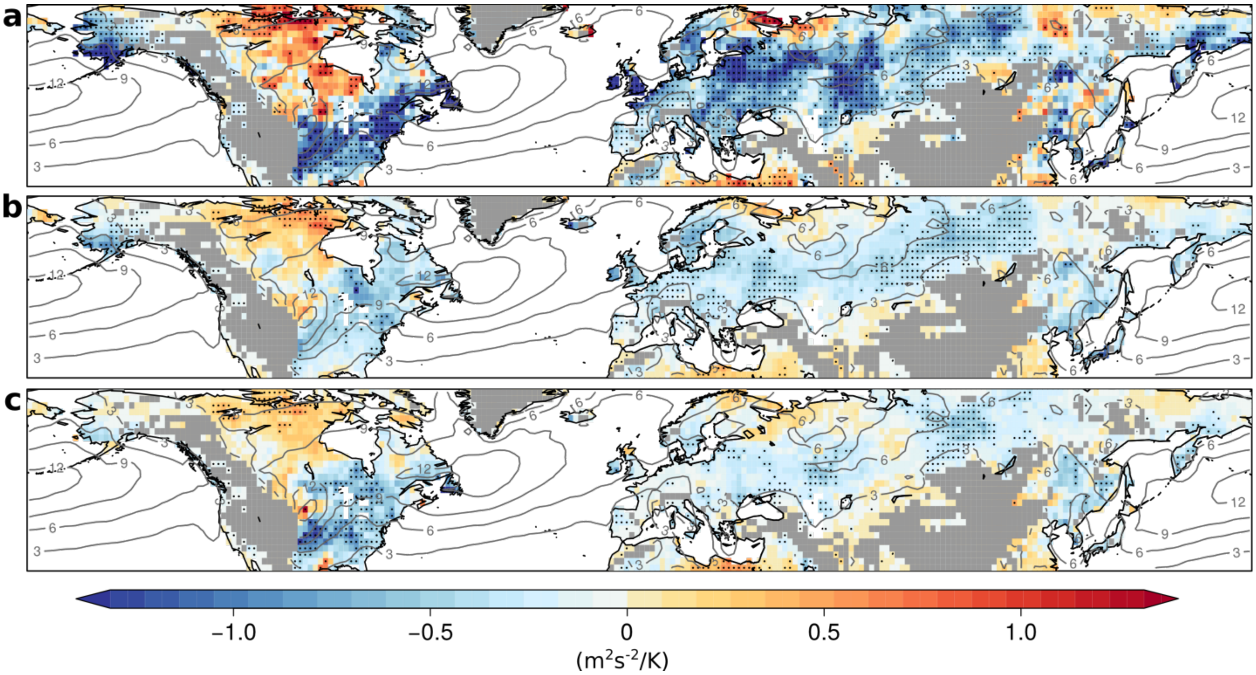

Fig. S3. Same as Fig. 1 in main manuscript but for quantile regression of v'^2 with summer temperatures. All maps are created using the open source software R.

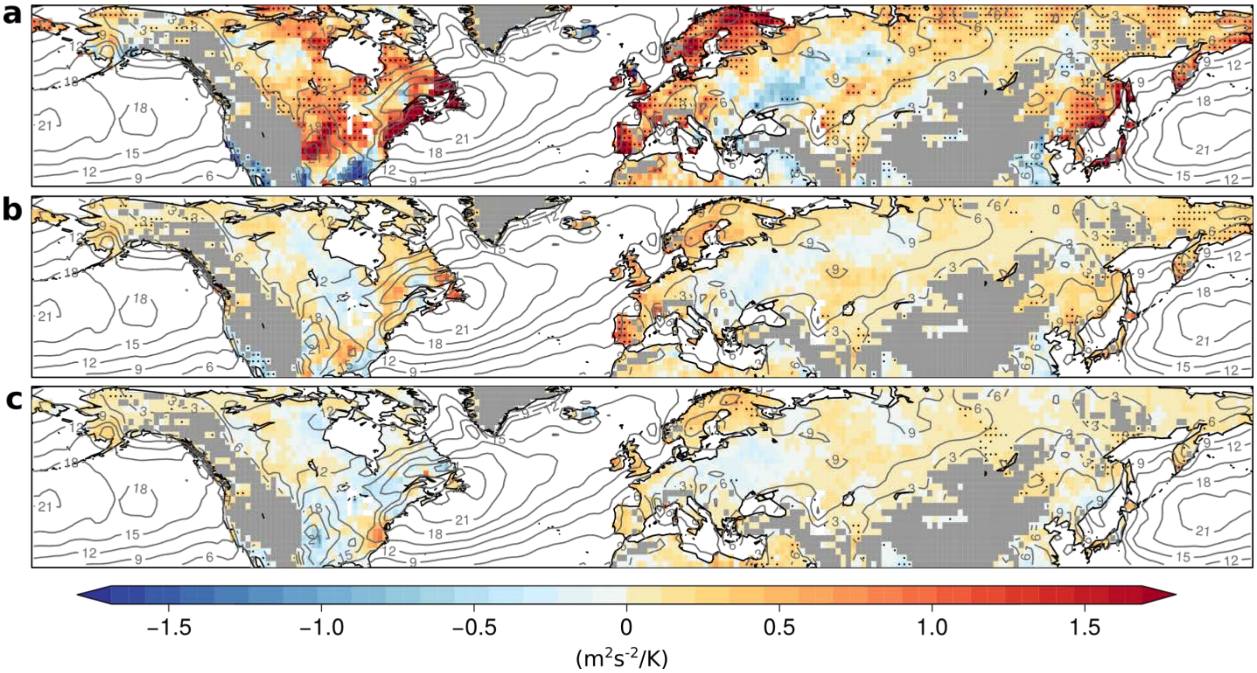

Fig. S4. Same as Fig. 2 in main manuscript but for quantile regression of v'^2 with winter temperatures. All maps are created using the open source software R.

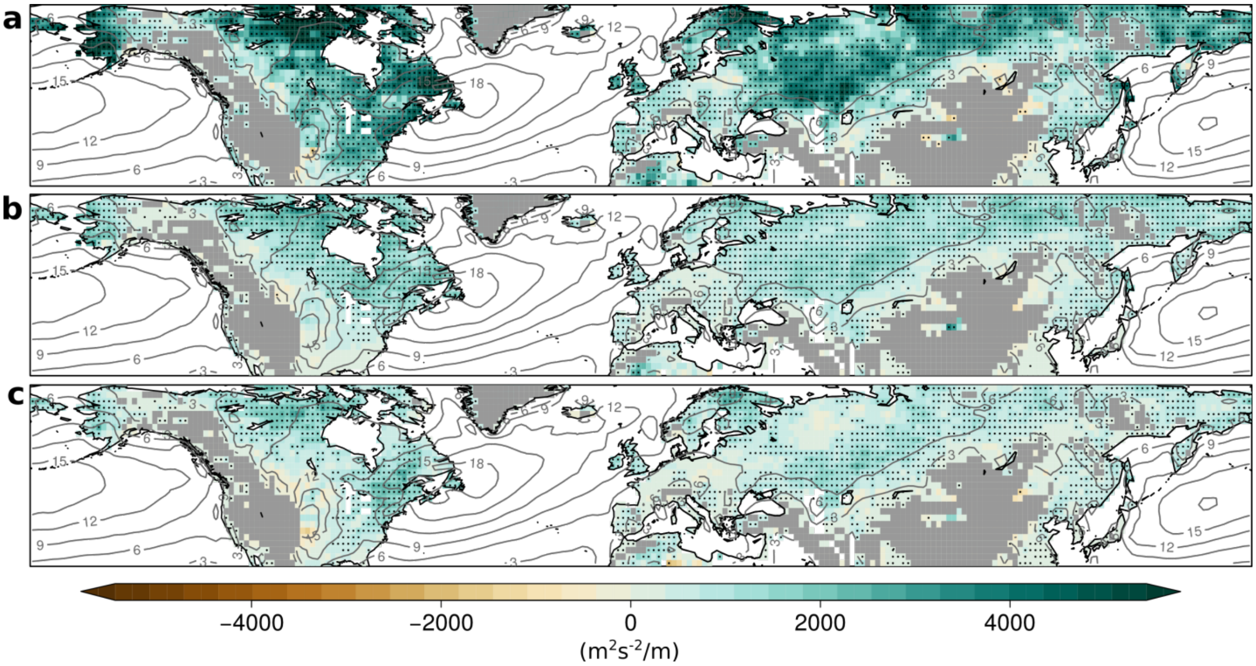

Fig. S5. Same as Fig. 3 in main manuscript but for quantile regression of v’^{2} with precipitation. All maps are created using the open source software R.

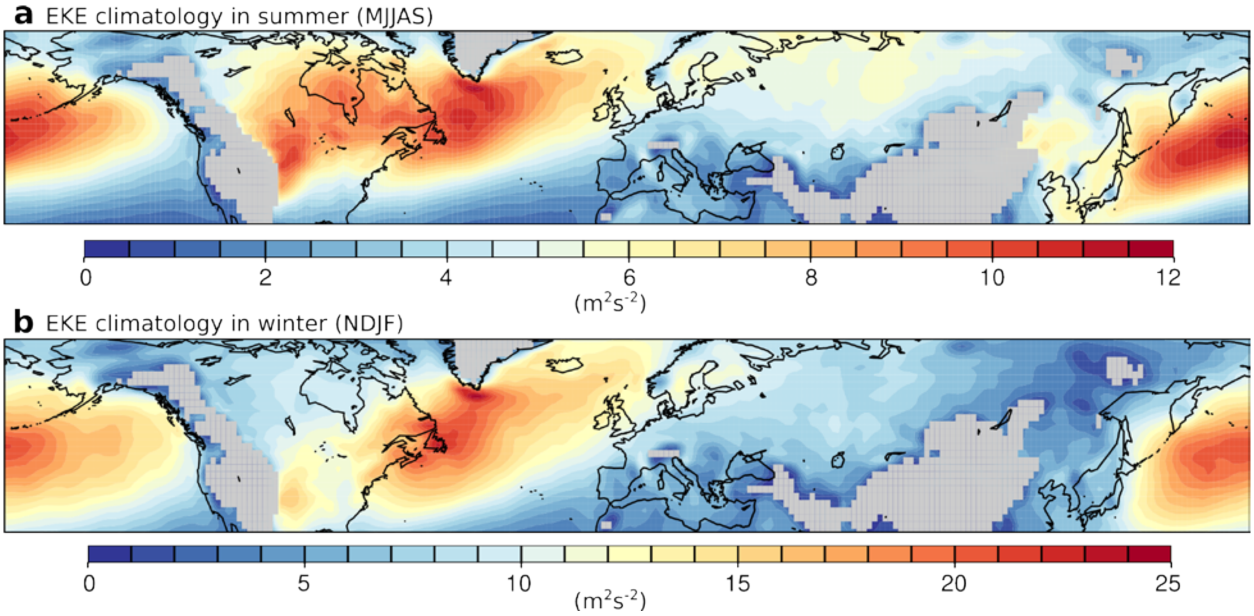

Fig. S6. EKE climatology at 850 mb. Contour plots of seasonally averaged EKE climatology at 850 mb computed for the full time period considered (1979-2014) and shown for May-June-July-August-September (**a**) and November-December-January-February (**b**). All maps are created using the open source software R.

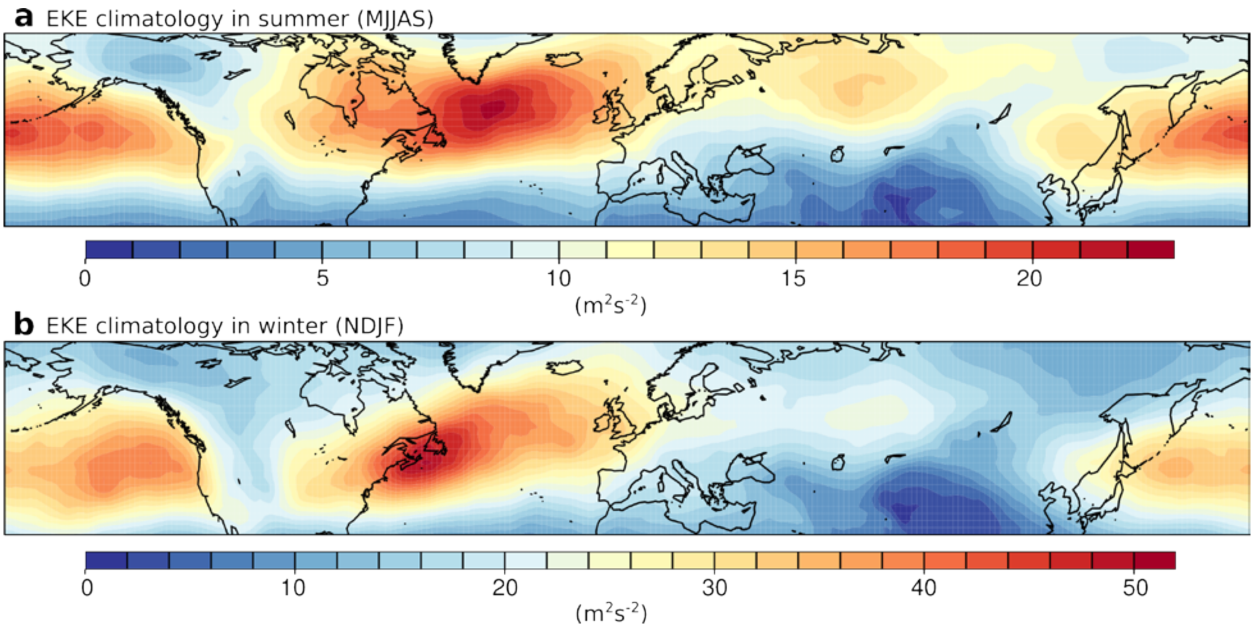

Fig. S7. EKE climatology at 500 mb. Contour plots of seasonally averaged EKE climatology at 500 mb computed for the full time period considered (1979-2014) and shown for May-June-July-August-September (**a**) and November-December-January-February (**b**). All maps are created using the open source software R.

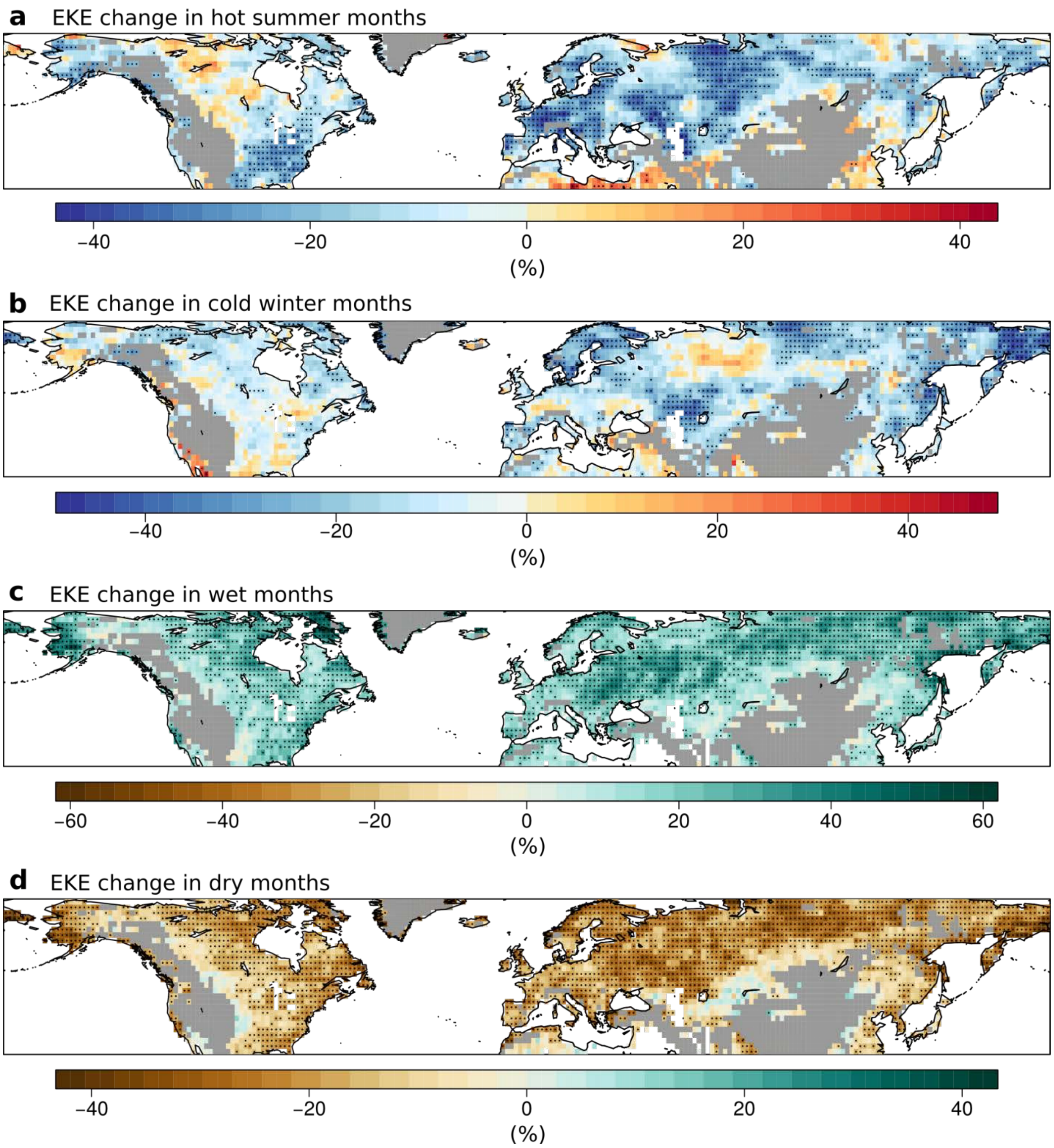

Fig. S8. Change in EKE in extreme months compared to local climatology. a-d, EKE in 10% hottest summer months (**a**), 10% coldest winter months (**b**), 10% wettest months (**c**), and 10% driest months (**d**) given as percentage change compared to local EKE climatology of given season. Stippling indicates significance at the 5%-level derived from a two-sample t-test. Land regions higher than 1 km have been masked. All maps are created using the open source software R.

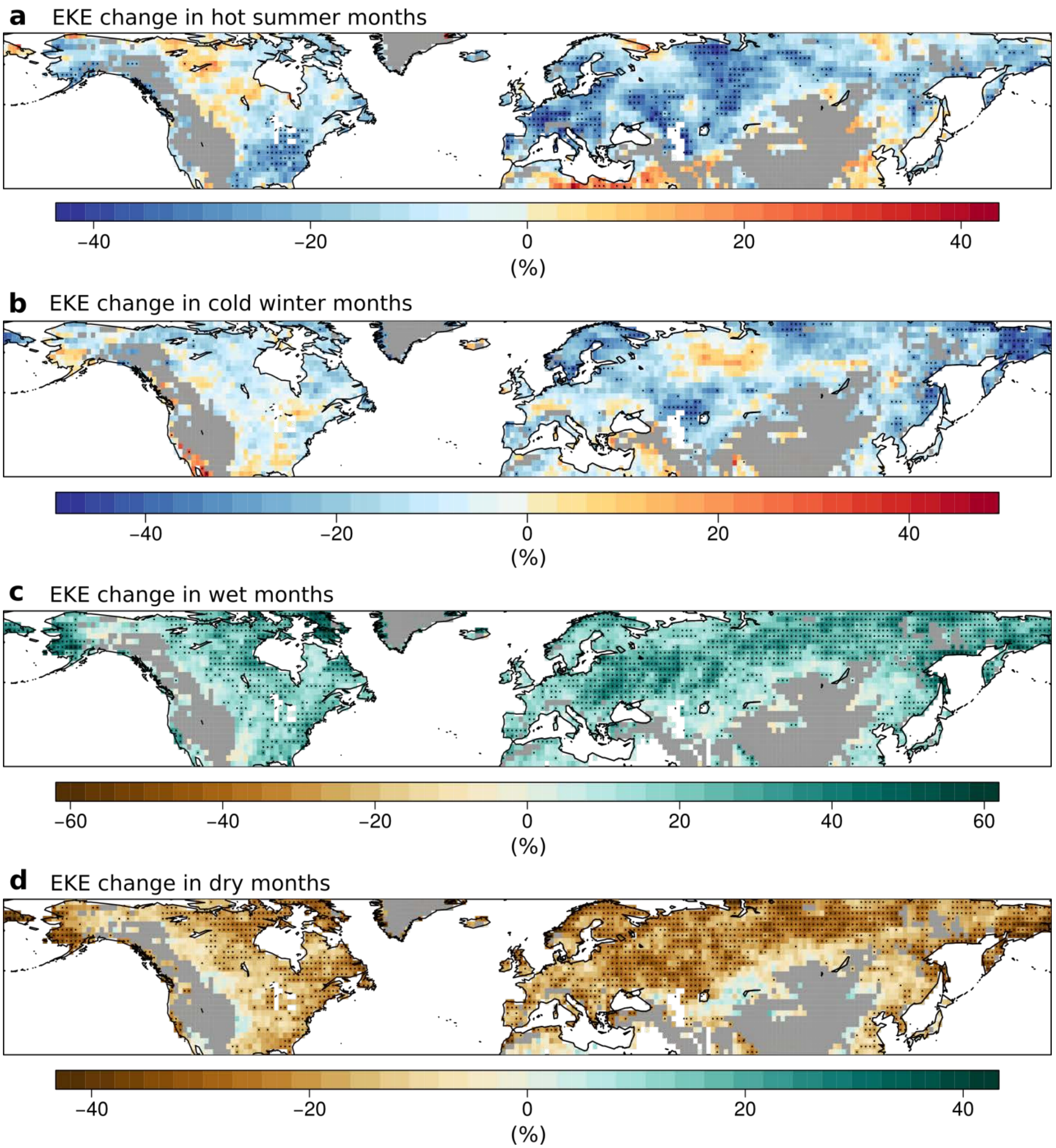

Fig. S9. Change in EKE in extreme months compared to climatology. Same as Fig. S6 but using Kolmogorow-Smirnow test to estimate significance at the 5%-level. Significant changes are found over similar regions as determined using a two-sample t-test (Fig. S6). All maps are created using the open source software R.

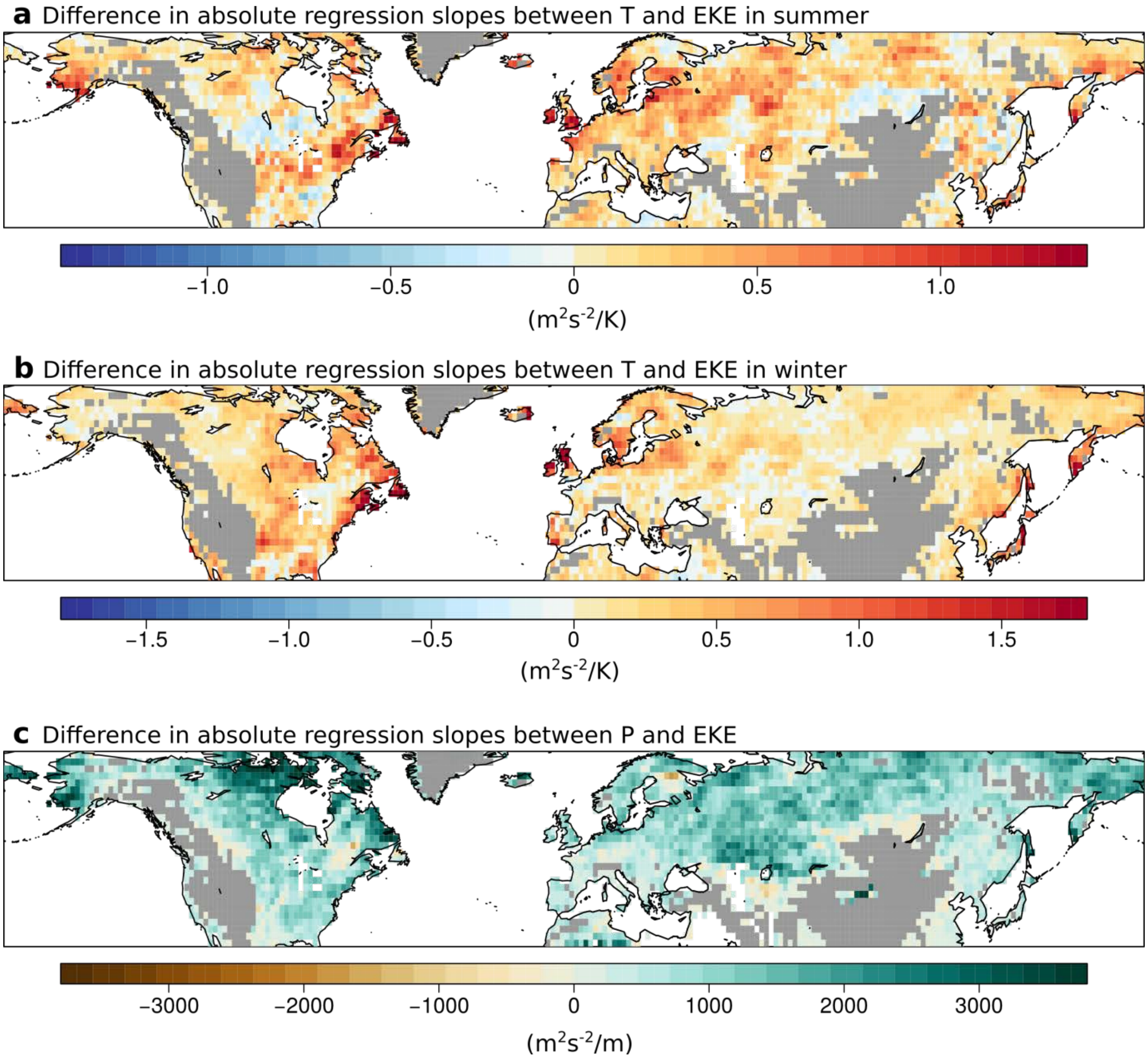

Fig. S10. Difference in absolute regression slopes between 90th and 10th-percentile. Results are shown for regressing EKE with temperatures in summer (**a**) and winter (**b**), and for regression analysis between annual EKE and precipitation (**c**). The difference was computed from absolute values of the regression slopes so that positive values in close to all regions in all three panels imply that regression slopes are steeper for higher quantiles. Land regions higher than 1 km have been masked. All maps are created using the open source software R.

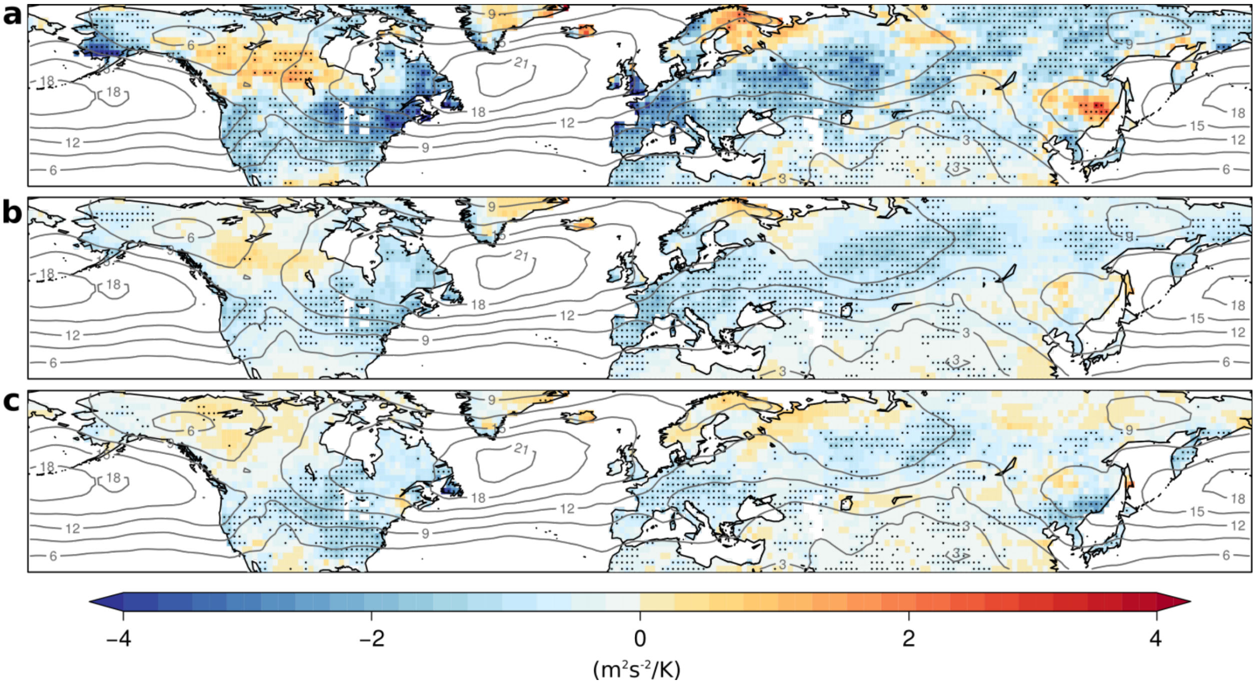

Fig. S11. Slope of quantile regressions between anomalies of EKE at 500 mb and temperature in summer. Same as Fig. 1 in main manuscript but for EKE at 500 mb. All maps are created using the open source software R.

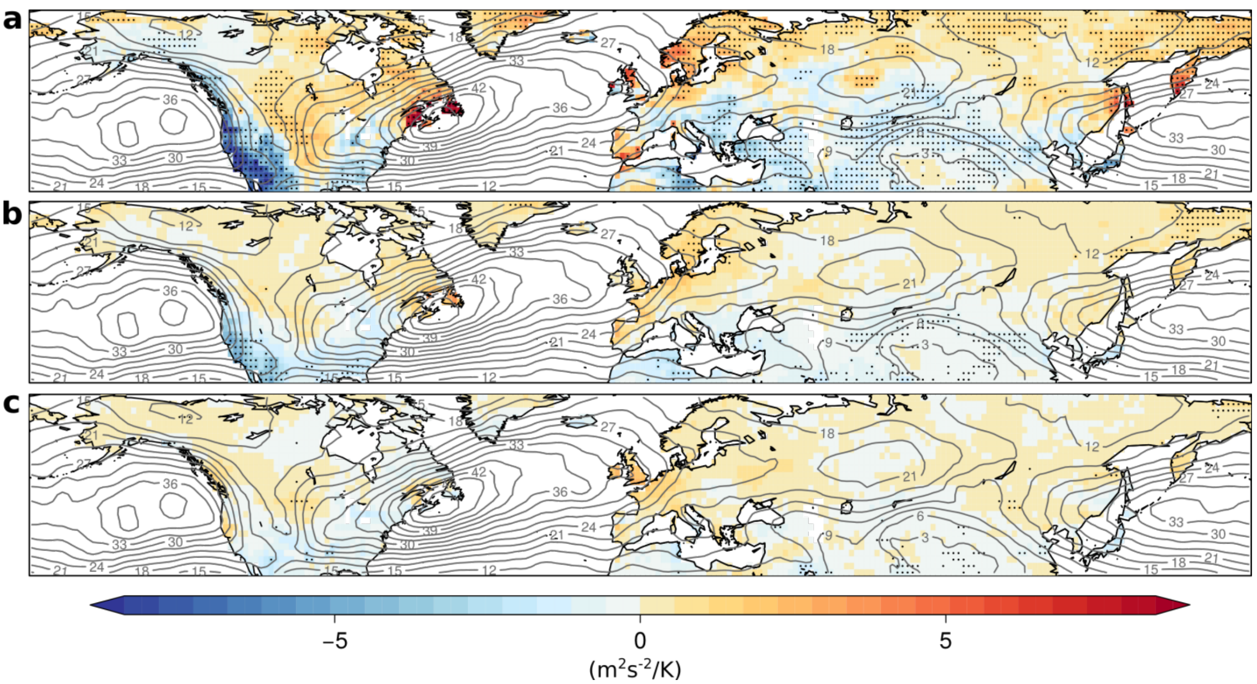

Fig. S12. Slope of quantile regressions between anomalies of EKE at 500 mb and temperature in winter. Same as Fig. 2 in main manuscript but for EKE at 500 mb. All maps are created using the open source software R.

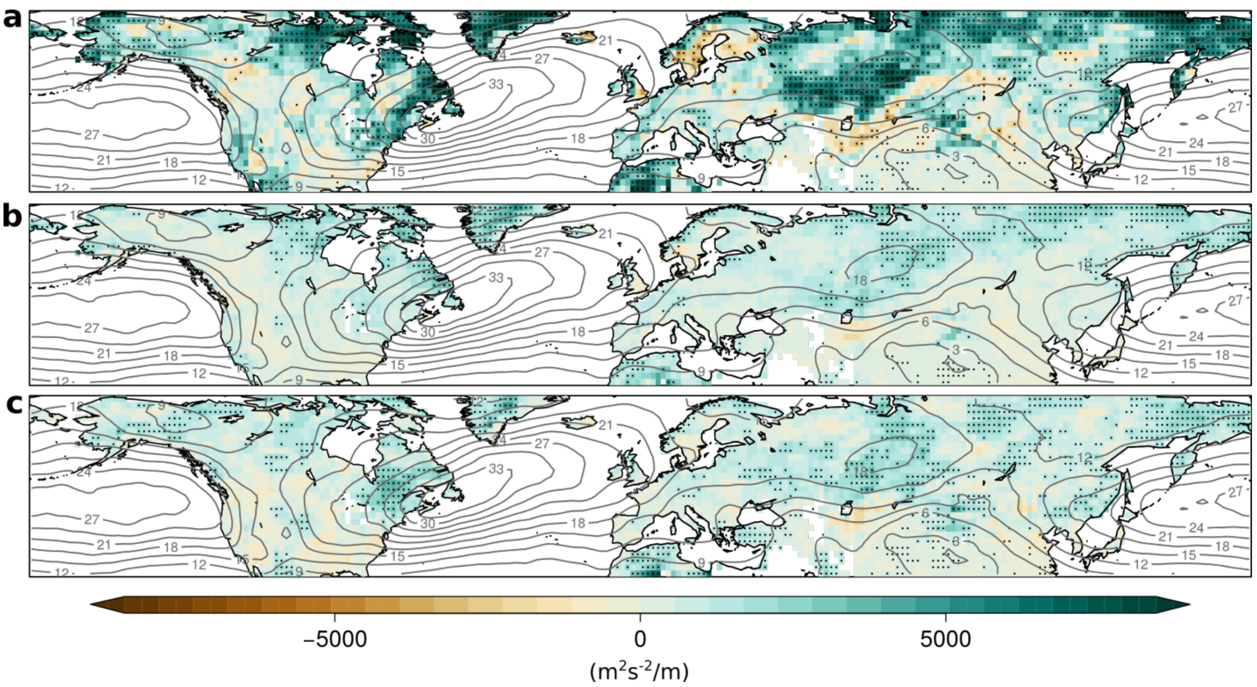

Fig. S13. Slope of quantile regressions between anomalies of EKE at 500 mb and precipitation in all calendar months. Same as Fig. 3 in main manuscript but for EKE at 500 mb. All maps are created using the open source software R.

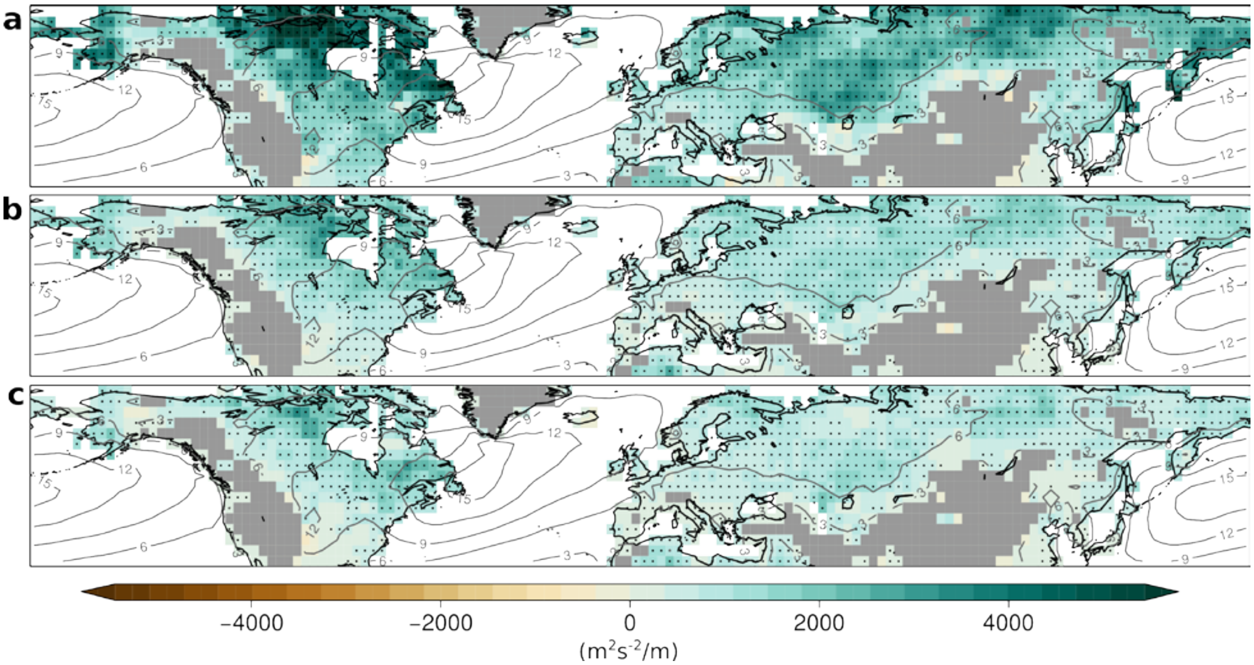

Fig. S14. Slope of quantile regressions between anomalies of EKE (at 850 mb) and precipitation (GPCP) in all calendar months. Same as Fig. 3 in main manuscript but with precipitation time series taken from the GPCP data set. All maps are created using the open source software R.

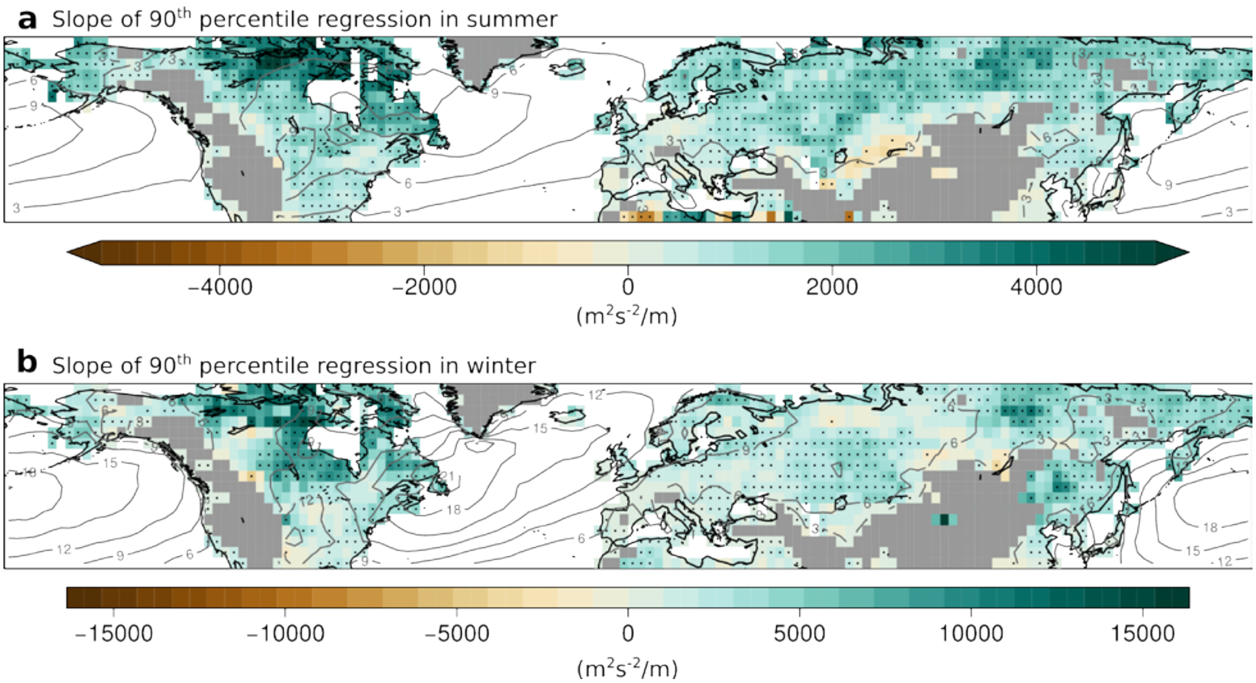

Fig. S15. Slope of 90th-percentile regressions between anomalies in EKE (at 850 mb) and precipitation (GPCP) in different seasons. Same as Fig. S2 but with precipitation time series taken from the GPCP data set. All maps are created using the open source software R.

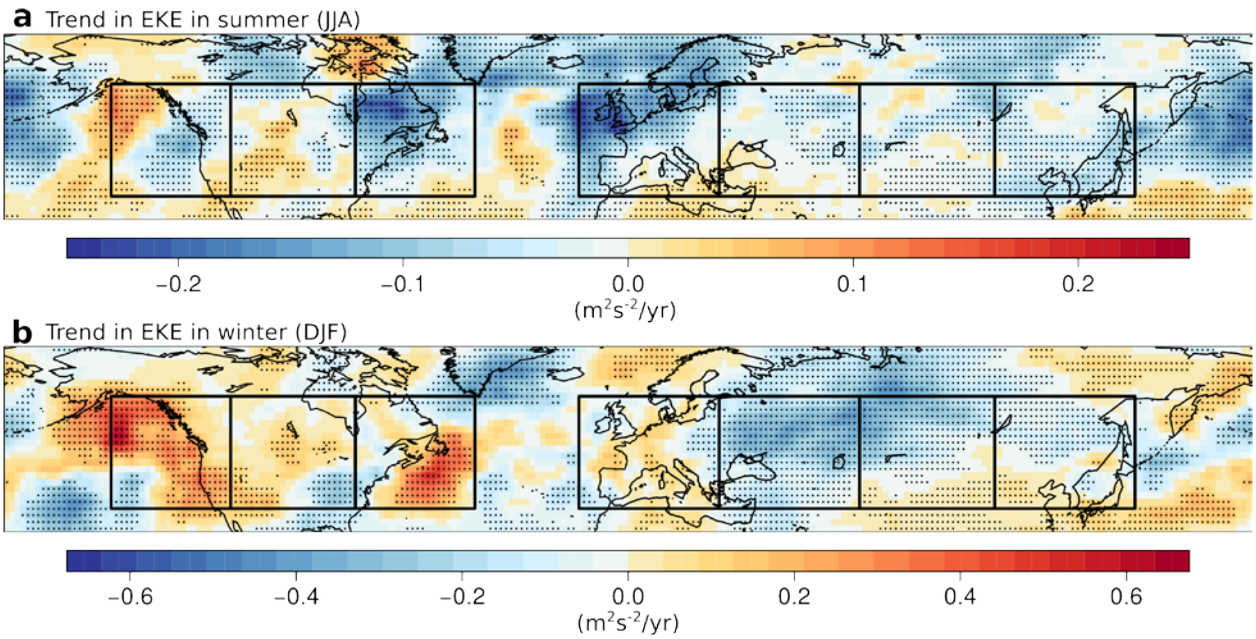

Fig. S16. Trends in EKE at 500 mb. Same as Fig. 5a, b in main manuscript but at 500 mb and seasonal averages are computed for June-July-August (**a**) and December-January-February (**b**) to make the figures comparable to results from Horton et al.[1]. Black boxes indicate the following regions used in Horton et al.[1]: (from left to right) eastern US, central US, eastern US, Europe, western Asia, central Asia, eastern Asia. All maps are created using the open source software R.

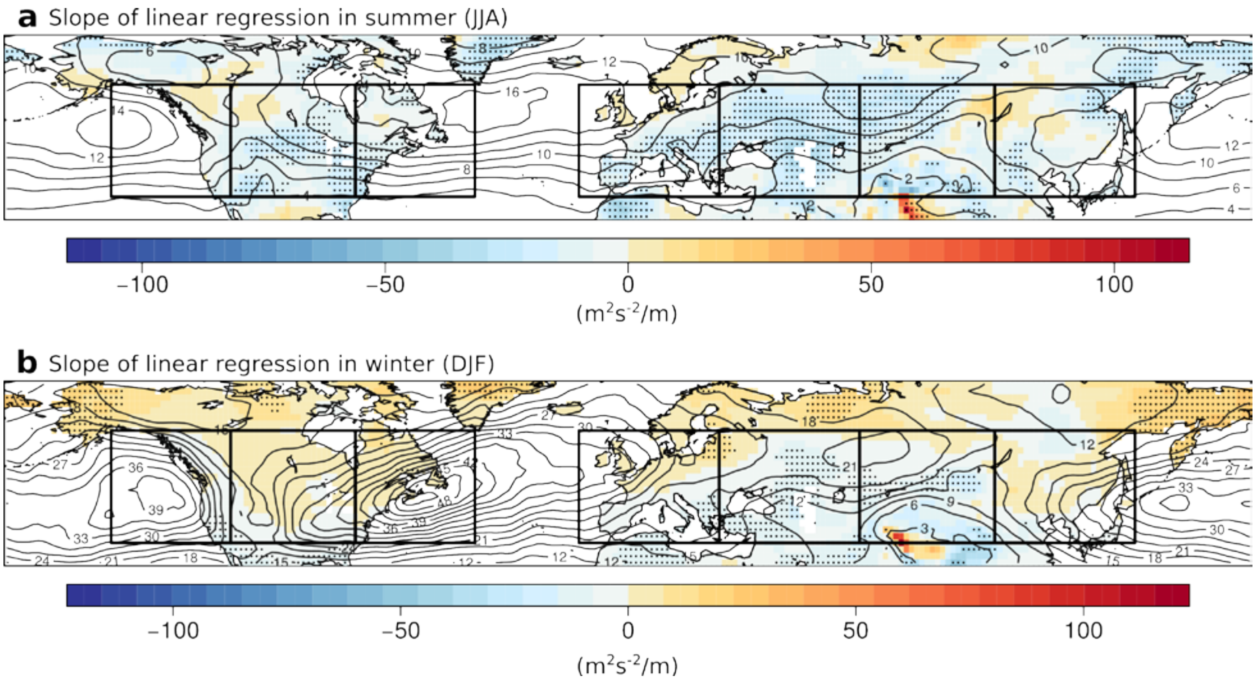

Fig. S17. Slope of linear regression between anomalies of EKE and GPH at 500 mb. Same as Fig. 4 in main manuscript but at 500 mb and seasonal averages are computed for June-July-August (**a**) and December-January-February (**b**) to make the figures comparable to results from Horton et al.[1]. Black boxes indicate the following regions used in Horton et al.[1]: (from left to right) eastern US, central US, eastern US, Europe, western Asia, central Asia, eastern Asia. All maps are created using the open source software R.

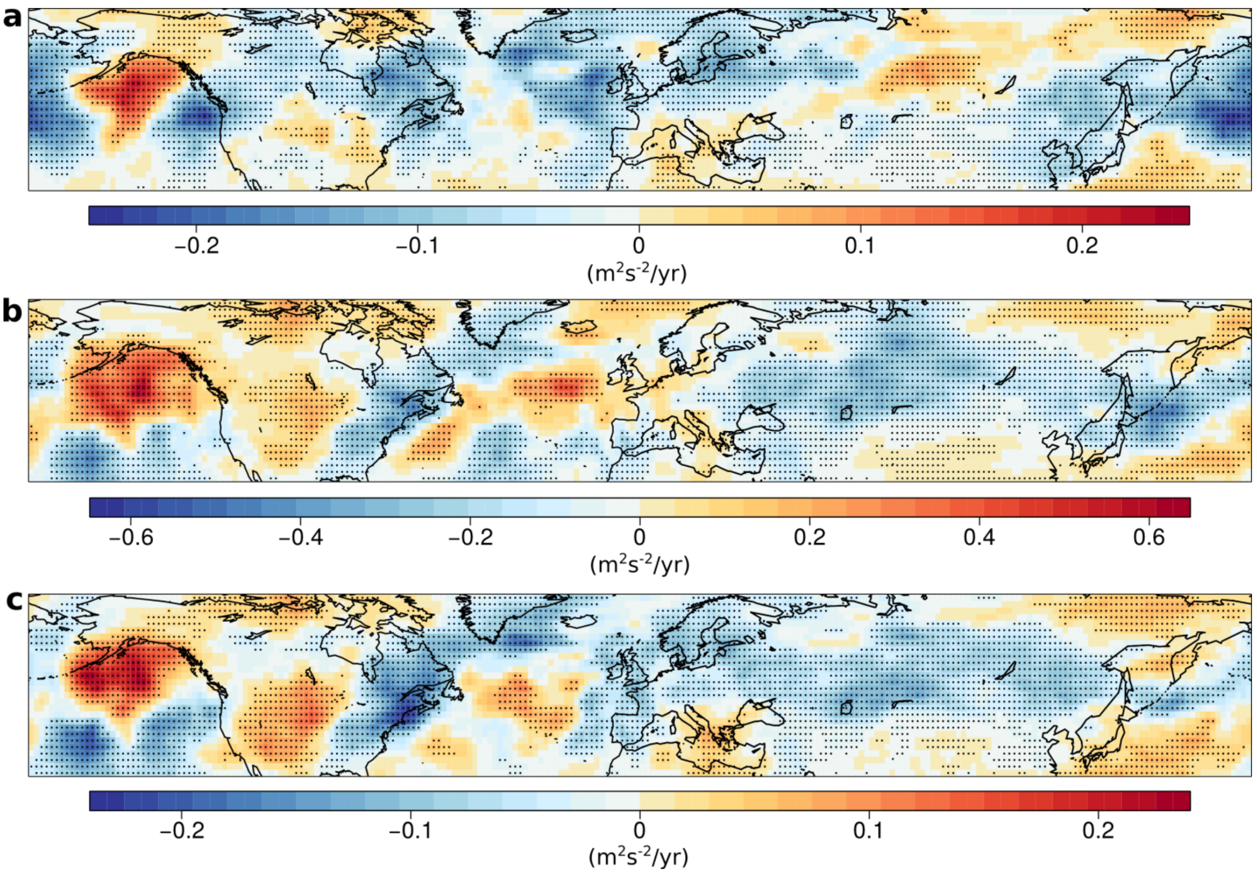

Fig. S18. Trends in EKE at 500 mb. Same as Fig. 5 in main manuscript but for EKE at 500 mb. All maps are created using the open source software R.

References used in SI

1. Horton, D. E. *et al.* Contribution of changes in atmospheric circulation patterns to extreme temperature trends. *Nature* **522,** 465–469 (2015).
